# Ultralow‐power in‐memory computing based on ferroelectric memcapacitor network

**DOI:** 10.1002/EXP.20220126

**Published:** 2023-05-11

**Authors:** Bobo Tian, Zhuozhuang Xie, Luqiu Chen, Shenglan Hao, Yifei Liu, Guangdi Feng, Xuefeng Liu, Hongbo Liu, Jing Yang, Yuanyuan Zhang, Wei Bai, Tie Lin, Hong Shen, Xiangjian Meng, Ni Zhong, Hui Peng, Fangyu Yue, Xiaodong Tang, Jianlu Wang, Qiuxiang Zhu, Yachin Ivry, Brahim Dkhil, Junhao Chu, Chungang Duan

**Affiliations:** ^1^ Key Laboratory of Polar Materials and Devices, Ministry of Education, Shanghai Center of Brain‐inspired Intelligent Materials and Devices, Department of Electronics East China Normal University Shanghai China; ^2^ Zhejiang Lab Hangzhou China; ^3^ School of Materials Science and Engineering Shanghai University of Engineering Science Shanghai China; ^4^ CentraleSupélec, CNRS‐UMR8580, Laboratoire SPMS Université Paris‐Saclay Gif‐sur‐Yvette France; ^5^ State Key Laboratory of Infrared Physics, Chinese Academy of Sciences Shanghai Institute of Technical Physics Shanghai China; ^6^ Frontier Institute of Chip and System Fudan University Shanghai China; ^7^ Institute of Optoelectronics Fudan University Shanghai China; ^8^ Guangdong Provisional Key Laboratory of Functional Oxide Materials and Devices Southern University of Science and Technology Shenzhen China; ^9^ Department of Materials Science and Engineering Solid‐State Institute Technion‐Israel Institute of Technology Haifa Israel; ^10^ Collaborative Innovation Center of Extreme Optics Shanxi University Shanxi China

**Keywords:** ferroelectric, in‐memory computing, memcapacitor, P(VDF‐TrFE), ultralow power

## Abstract

Analog storage through synaptic weights using conductance in resistive neuromorphic systems and devices inevitably generates harmful heat dissipation. This thermal issue not only limits the energy efficiency but also hampers the very‐large‐scale and highly complicated hardware integration as in the human brain. Here we demonstrate that the synaptic weights can be simulated by reconfigurable non‐volatile capacitances of a ferroelectric‐based memcapacitor with ultralow‐power consumption. The as‐designed metal/ferroelectric/metal/insulator/semiconductor memcapacitor shows distinct 3‐bit capacitance states controlled by the ferroelectric domain dynamics. These robust memcapacitive states exhibit uniform maintenance of more than 10^4^ s and well endurance of 10^9^ cycles. In a wired memcapacitor crossbar network hardware, analog vector‐matrix multiplication is successfully implemented to classify 9‐pixel images by collecting the sum of displacement currents (*I* = *C* × d*V*/d*t*) in each column, which intrinsically consumes zero energy in memcapacitors themselves. Our work sheds light on an ultralow‐power neural hardware based on ferroelectric memcapacitors.

## Graphical abstract

We demonstrate a robust memcapacitor by stacking ferroelectric capacitor on the metal‐insulator‐semiconductor structure. It possesses a long retention time of more than 10^4^ s and well endurance of 10^9^ cycles. Ultralow‐power in‐memory computing based on ferroelectric memcapacitor array intrinsically consumes zero energy in memcapacitors themselves. It sheds light on an ultralow‐power neural hardware based on ferroelectric memcapacitors.

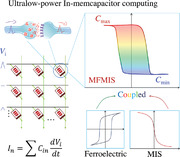

## 1 INTRODUCTION

Inspired by the biological brain, neuromorphic computing has seen a resurgence of interest for the advantages it offers in terms of high energy efficiency, massive parallelism, and adaptive learning features.^[^ ^1^, ^2^, ^3^, ^4^, ^5^ ^]^ The development of memristive devices has promoted the implementation of neuromorphic computing at the hardware level.^[^ ^6^, ^7^ ^]^ The reported memristors involve oxide resistive memories,^[^ ^8^, ^9^, ^10^ ^]^ phase‐change memories,^[^ ^11^ ^]^ spintronic and ferroelectric memories.^[^ ^12^, ^13^, ^14^, ^15^, ^16^ ^]^ All these devices rely on the analog synaptic weights through conductance states, which are used in vector‐matrix multiplication (VMM) operations along Ohm's and Kirchhoff's laws, hence inevitably generate harmful heat dissipation.^[^ ^1^ ^]^ In addition, along with the reduction in device size, thermal noises in such current‐powered devices also undoubtedly degrade the operation of the crossbar‐based array.

Memcapacitive devices, based on a capacitive principle, potentially offer a lower power consumption since the information is transmitted in the form of charges rather than currents. Various strategies have been tried to obtain the memcapacitive memory, such as the modulation of the effective area (permittivity) of a capacitor,^[^ ^17^, ^18^ ^]^ the charge shielding,^[^ ^19^ ^]^ the ions migration,^[^ ^20^ ^]^ the insertion of a memristor in series with a capacitor,^[^ ^21^ ^]^ the charge injection and recovery,^[^ ^22^ ^]^ and so on. Although several synaptic applications have progressed in the aforementioned memcapacitors, memcapacitive neural networks are at a very preliminary stage in terms of potential practical applications and there is not even a consensus yet on how to operate at the hardware level.

Here, we report robust memcapacitive behavior within a simple stacked structure of a ferroelectric polymer capacitor on a SiO_2_/Si substrate. Both metal/ferroelectric/insulator/semiconductor (MFIS) and metal/ferroelectric/metal/insulator/semiconductor (MFMIS) structures are fabricated and show reversible multi‐level capacitance states that are explicitly controlled by the ferroelectric domain dynamics. More strikingly, the sandwiched metal layer in between the ferroelectric and the insulator in MFMIS structures rearranges the induced charges to achieve a uniform‐field modulation on the semiconductor, resulting in reconfigurable intermediate capacitance states with a long lifetime of more than 10^4^ s and a good endurance of 10^9^ cycles. The electrical formula of *I* = *C* × d*V*/d*t* is then used to perform analog VMM operations in crossbar network hardware made with the above memcapacitors, wherein *C* is the variable capacitance state enabled by the memcapacitor, and triangular voltage waves with different d*V*/d*t* are encoded as input signals. As an illustration, a wired ferroelectric memcapacitor crossbar network hardware is used to successfully classify 9‐pixel images.

## 2 RESULTS AND DISCUSSION

### 2.1 Mechanism behind the ferroelectric‐based memcapacitor

**FIGURE 1:**
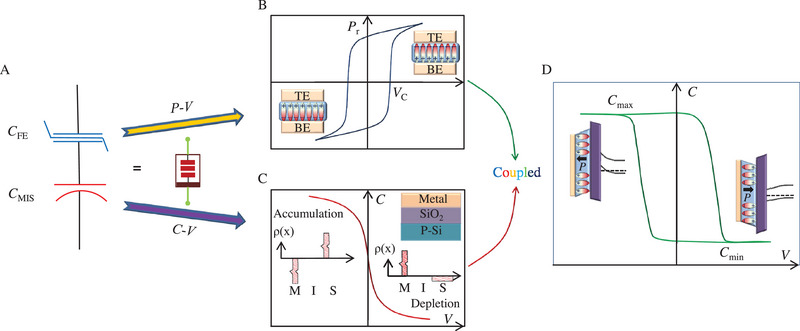
The designed ferroelectric memcapacitor and its *C*–*V* property. A) Configuration of polarization‐controlled memcapacitor with a stacked structure of a ferroelectric capacitor and a metal/insulator/semiconductor (MIS) structure. B) The typical *P*–*V* hysteresis loop of a ferroelectric capacitor. Insets show ferroelectric domains after positive (negative) voltage bias that is larger than positive (negative) coercive voltage (*V*_C_). C) The *C*–*V* curve of an MIS varactor. Insets show the structure of the MIS capacitor, and the charges distribution under negative and positive voltage, respectively. D) The *C*–*V* hysteresis loop of the metal/ferroelectric/insulator/semiconductor (MFIS) structure. Insets show the ferroelectric domains and the energy band alignment after positive (negative) voltage bias that is larger than positive (negative) *V*_C_.

A stacked structure of a ferroelectric capacitor in series with a metal/insulator/semiconductor (MIS) varactor is designed as a ferroelectric memcapacitor (Figure 1A). The spontaneous polarization in ferroelectrics can be switched reversibly and in a non‐volatile manner through the typical polarization versus voltage (*P*–*V*) hysteresis loop (Figure 1B). Multiple intermediate ferroelectric domain configurations during the polarization switching allow possible analog modulation of the capacitance when the ferroelectric layer is coupled to an MIS varactor. Generally, the mechanism of an MIS varactor can be easily understood using a simple parallel‐plate capacitor. The capacitance of an MIS varactor can be described as: $C_{\mathrm{MIS}}=\frac{\varepsilon_{\mathrm{ox}}S}{t_{\mathrm{ox}}\;+\;\left(\frac{\varepsilon_{\mathrm{ox}}}{\varepsilon_{s}}\right)t_{d}}$, where ε_ox_ and *t*_ox_ are the permittivity and effective thickness of the insulator (which is usually an oxide) layer, *S*, ε_s_, and *t*_d_ are the area, permittivity and effective thickness of the depleted layer in the semiconductor, respectively.^[^ ^23^ ^]^ Figure 1C displays the capacitance versus voltage (*C*–*V*) curve of an MIS varactor with a p‐type Si semiconductor and silicon oxide as the insulator layer. The voltage is applied to the metal with the semiconductor grounded. Under a negative voltage bias, the majority‐carrier holes would accumulate at the insulator/semiconductor interface, resulting in a negligible *t*_d_ and a large *C*_MIS_ value. In contrast, a positive voltage bias would deplete majority‐carrier holes at the insulator/semiconductor interface, leading to *t*_d_ that increases gradually with voltage increase. A larger *t*_d_ corresponds to a smaller value of *C*_MIS_ until the inversion of the majority‐carrier type, by an ultrahigh positive voltage. Notably, the capacitance in a single MIS varactor varies in a volatile manner. By combining a ferroelectric top layer with an MIS varactor to form an MFIS structure, multi‐level capacitances are therefore accessible since the nonvolatile ferroelectric domain states can provide, thanks to their polarization, pure bound charges at the ferroelectric/insulator interface. In turn, these bound charges produce a strong local electric field to the semiconductor, acting similarly to an external voltage bias. The capacitance versus voltage (*C*–*V*) hysteresis loop in an MFIS structure is displayed in Figure 1D. The accumulation and depletion of carriers in the underneath p‐type semiconductor induced by the upward and downward ferroelectric polarizations give rise to high (ON) and low (OFF) capacitance states, respectively.

### 2.2 Memcapacitive behavior in the MFIS memcapacitor

**FIGURE 2:**
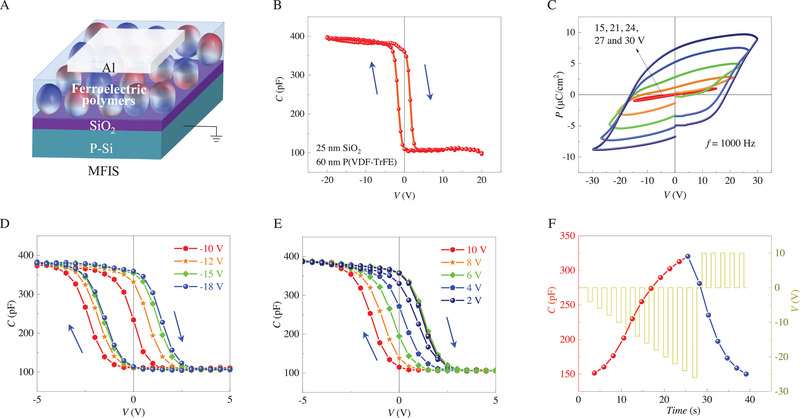
The ferroelectric memcapacitor with a metal/ferroelectric/insulator/semiconductor (MFIS) structure. A) The sketch of the MFIS memcapacitor. B) *C*–*V* hysteresis loop of the MFIS memcapacitor with 60 nm‐thick ferroelectric polymers and 25 nm‐thick SiO_2_. C) *P*–*V* hysteresis loops of Al/P(VDF‐TrFE)/Al capacitor with voltage amplitudes of 15, 21, 24, 27, and 30 V, respectively. The measured frequency is 1 kHz. D) *C*–*V* hysteresis loops of the MFIS memcapacitor with a constant positive voltage amplitude of 10 V and negative voltage amplitudes of − 10,− 12,− 15, and − 18 V, respectively. E) *C*–*V* hysteresis loops of the MFIS memcapacitor with a constant negative voltage amplitude of − 18 V and positive voltage amplitudes of 2, 4, 6, 8, and 10 V, respectively. F) Evolution of capacitance on the negative voltage pulse sequence with an amplitude gradually increasing from − 4 to − 26 V and a width of 1 s (interval 1 s) followed by the positive voltage pulse sequence with a constant amplitude of + 10 V and a width of 1 s (interval 1 s).

Figure 2A presents the fabricated ferroelectric memcapacitor in which the top Al electrode, the ferroelectric poly(vinylidene fluoride) copolymer P(VDF‐TrFE) (70:30 mol%) and the insulator layer (SiO_2_) are vertically stacked on a p‐type Si semiconductor to form the MFIS structure. The surface of P(VDF‐TrFE) films in our work exhibits grain‐like morphology with a root‐mean‐square roughness of about 2.59 nm as shown in Figure S1A. To check the ferroelectricity, local piezoresponse force microscopy (PFM) is performed with the bottom electrode being grounded. Clear ferroelectric signals with 180°‐contrast phase and butterfly amplitude are observed in these P(VDF‐TrFE) thin films (Figure S1B). Then a square region followed by a smaller “heart” region is biased through the conductive tip with + 20 and − 20 V, respectively. Voltage bias‐induced ferroelectric domain reversals are confirmed by both 180°‐contrast in phase image (Figure S1C) and low‐lying boundary in amplitude image (Figure S1D).

The single stacked MFIS structure allows us to consider the memcapacitance (*C*_m_) as a serial combination of the ferroelectric capacitor (*C*_F_) and the insulator/semiconductor capacitor (*C*_IS_), which can be expressed using an equivalent electrical circuit model, by $\frac{1}{C_{m}}=\frac{1}{C_{F}}\;+\;\frac{1}{C_{\mathrm{IS}}}$. Here the *C*_IS_ is actually the same as *C*_MIS_ mentioned above. Within this model, we can estimate the contribution of each individual capacitor to the memory effect. Typically, ferroelectrics possess a butterfly‐like *C*–*V* loop under a bipolar electric field. There is a minor increment (< 50%) of capacitance with applied voltage sweeping from one direction to the other one. Although such characteristics were proposed in Zheng's work for artificial neural networks (ANNs),^[^ ^18^ ^]^ it requires complicated circuits and reset operation that are not suitable for application in memcapacitive crossbar arrays. In our MFIS structure, besides the capacitance variation of the ferroelectric under voltage solicitation, the *C*_IS_ can be effectively modulated via the remnant polarization of the ferroelectric. The precise control of the polarization can be realized because the domain configuration in ferroelectric materials can be modulated by tuning the characteristics (magnitude, width, and/or frequency) of applied voltage pulses.^[^ ^24^, ^25^, ^26^ ^]^ Such domain arrangements of the ferroelectric hence lead to the multiple capacitance states, that is, memcapacitor behavior, in our MFIS structure. The SiO_2_ insulator is indispensable here as a buffer layer to neutralize the influence of dangling bonds at the Si surface.

Figure 2B shows *C*–*V* hysteresis loop of the MFIS memcapacitor with 60 nm‐thick ferroelectric polymer layer and 25 nm‐thick SiO_2_ layer. Directed evidence of the memcapacitance effect is obtained, where the capacitance is switched hysteretically between OFF and ON states by the bipolar voltage applied to the top electrode. The coupling effect between ferroelectric polarization and dynamics of semiconductor carriers is inferred from the clockwise capacitance hysteresis. The two distinct capacitances at *V* = 0 V arise from electric charges at the insulator/semiconductor interface induced by the nonvolatile ferroelectric polarization. When a high positive voltage is applied, P(VDF‐TrFE) is totally polarized downward and the holes are depleted at the SiO_2_/p‐Si interface, corresponding to a minimum capacitance value. Then when the voltage sweeps from + 20 to − 20 V, the capacitance remains unchanged until the negative voltage reaches a threshold value, which drives the polarization reversal and hole accumulation that finally results in the highest capacitance. A similar transition behavior is observed while sweeping the voltage backward.

In addition to PFM (Figure S1), the ferroelectricity of the P(VDF‐TrFE) thin films is further confirmed by *P*–*V* hysteresis loops of an Al/P(VDF‐TrFE) (∼ 150 nm)/Al capacitor (Figure 2C). Multiple inner hysteresis loops can be obtained by gradually decreasing the amplitude of the sweeping voltage, suggesting a memcapacitance behavior is expected in the MFIS structure. To confirm it, *C*–*V* hysteresis loops with a constant positive voltage amplitude of 10 V and negative voltage amplitudes of − 10,− 12,− 15, and − 18 V, respectively (Figure 2D), are measured in the MFIS stack. Intermediate capacitance states across 0 V are clearly observed. Larger negative amplitudes lead to higher intermediate capacitance values. A similar behavior happens if fixing the negative amplitude and changing the positive amplitude (Figure 2E), with a lower capacitance value for a larger positive amplitude. Note that the negative shift of the hysteresis loop may originate from the difference of work function between Al metal (∼ 4.28 eV) and p‐type Si semiconductor with the resistivity of 1 Ω•cm (∼ 5 eV), and from the existence of positive defect charges, such as residual impurities of Na^+^ and K^+^ ions, in SiO_2_ layer. Both features would generate positive electric field in the semiconductor even without the application of an external voltage.

This memcapacitive, persistent, and multilevel programming of capacitance by the ferroelectric polarization can be used to emulate the synaptic weights. By specifically programming the negative and positive pulses, the capacitance value can be reversibly modulated in a quasi‐linear way (Figure 2F), implying that this memcapacitor shows promising potential for applications in ANNs. The retention behavior of the capacitance states in the MFIS memcapacitor is also tested. It is found that the capacitance value tends to gradually relax back with a time scale of few tenths of seconds (Figure S2). This depression of capacitance is probably related to the back‐switching of polarization due to the high internal electric field (*E*_int_) that originates from poor compensation for polarization charges at the ferroelectric/insulator interface.^[^ ^27^, ^28^ ^]^ To cure this effect, we fabricate an MFMIS structure by inserting in the MFIS stack a metal layer in between the ferroelectric and insulator.

### 2.3 Robust retention property in MFMIS memcapacitor

**FIGURE 3:**
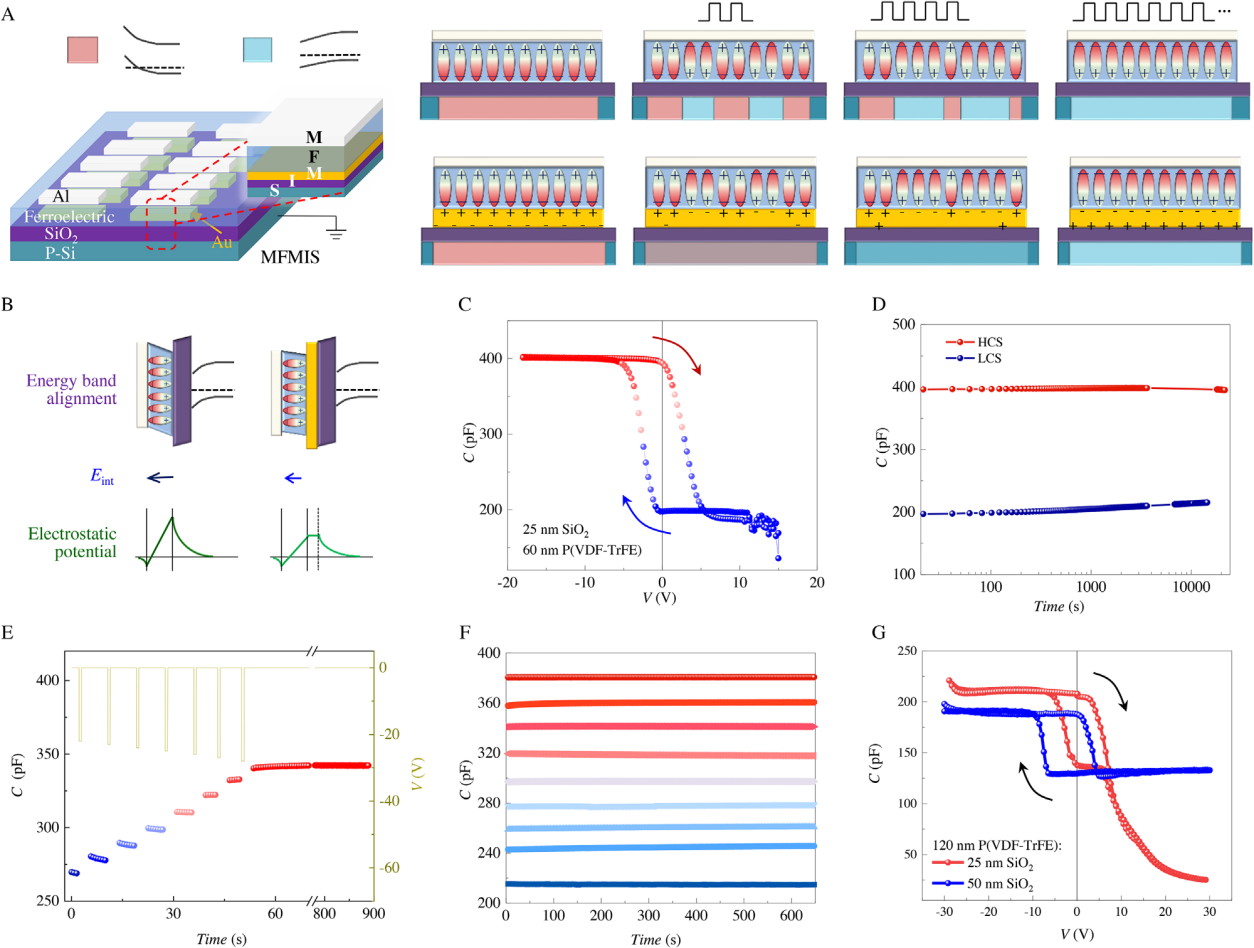
The ferroelectric memcapacitor with a metal/ferroelectric/metal/insulator/semiconductor (MFMIS) structure. A) Sketch of the MFMIS memcapacitor array. The right panel presents the evolution of ferroelectric domains and the modulation of carriers in the bottom p‐type Si semiconductor under positive voltage pulses. B) The steep electrostatic potential in metal/ferroelectric/insulator/semiconductor (MFIS) (highlighted by dark green) results in a huge *E*_int_ (highlighted by dark blue), while the softened electrostatic potential in MFMIS (highlighted by light green), by adequate charge compensation at the ferroelectric/metal interface and rearrangement of induced charges at the metal/insulator interface, results in a much smaller *E*_int_ (highlighted by light blue). C) *C*–*V* hysteresis loops of an MFMIS memcapacitor. D) Long‐time (> 10^4^ s) retention of highest capacitance state (HCS) and lowest capacitance state (LCS) in an MFMIS memcapacitor. E) Evolution of capacitance value on the negative voltage pulse sequence with a width of 1 s and amplitude of −22, −23, −24, −25, −26, −27, and −28 V, respectively. F) Robust retention property of 3‐bit capacitance states in an MFMIS memcapacitor. G) *C*–*V* hysteresis loops of the MFMIS memcapacitor with 120 nm‐thick P(VDF‐TrFE) and 25 nm‐thick (red) or 50 nm‐thick (blue) SiO_2_.

The MFIS memcapacitors have shown memcapacitive memory capability, and still could be applied for time‐permitted computing tasks. However, the nonvolatility remains essential for computing tasks such as convolution operations and transfer learning functions in an ANN hardware.^[^ ^29^ ^]^ To improve the retention performance of the intermediate capacitances observed in the MFIS, a metal layer is sandwiched between the ferroelectric and insulator layers to form an MFMIS structure (Figure 3A). In the case of the MFIS structure, that is, without the sandwiched metal layer, the polarization charges are not adequately compensated for both upward and downward domains, and an intermediate multi‐domain state gives utterly different modulation to the underneath semiconductor layer that depends on how the polarization of each domain is screened. On the contrary, the sandwiched metal layer in the MFMIS structure, that is, with the inserted metal layer, not only provides adequate charge compensation for all multi‐domains at the ferroelectric/metal interface, but also facilitates uniform modulation to the adjacent semiconductor by rearranging the induced charges at the metal/insulator interface. As also illustrated in Figure 3B, in addition to the uniformity of the charge modulation on the semiconductor, the appropriate charge compensation in the MFMIS triggers a much smaller *E*_int_ on the ferroelectric layer than that in MFIS. Once the *E*_int_ is lower than the coercive field of the ferroelectric, the intermediate multi‐domains can survive for a very long time. Remarkably, such extra metal layer also allows full utilization of inner *P*–*V* hysteresis loops (Figure 2C) and to achieve more intermediate states in the MFMIS memcapacitor.

Figure 3C displays *C*–*V* hysteresis loops of a typical MFMIS memcapacitor. Compared with MFIS, the MFMIS memcapacitor exhibits a smoother transition between ON and OFF states, and better maintains the capacitance state with only a very slight degradation after several hours (Figure 3D). The slower transition and longer retention time permit the various intermediate capacitance states to be nonvolatile. Figure 3E depicts the process of tuning an MFMIS memcapacitor from low capacitance to high capacitance states by gradually increasing the amplitude of negative voltage pulses. These memcapacitive states are measured after each pulse, revealing a step‐like nonvolatile memory. The last step is checked with time and shows no degradation for 15 min. Nine (> 2^3^) different memcapacitive states, evenly distributed between ON and OFF states, display good stability in the test period of more than 10 min (Figure 3F). Figure 3G shows *C*–*V* hysteresis loops of the MFMIS memcapacitor with 120 nm‐thick P(VDF‐TrFE) and 25 nm‐thick (red) or 50 nm‐thick (blue) SiO_2_, both of which reveal good retention as well (Figure S3). The reduction of the capacitance value and ON/OFF ratio for the larger thickness SiO_2_ layer (or of ferroelectric polymer layer) agrees well with the parallel‐plate model as mentioned previously. It should be noted that the abnormal decay of capacitance under high voltage range (above 10–15 V) in Figure 3C,G, despite a little influence on the *C*–*V* hysteresis loops, may imply other interesting physics.

### 2.4 Implementations of VMM operations and pattern recognition

**FIGURE 4:**
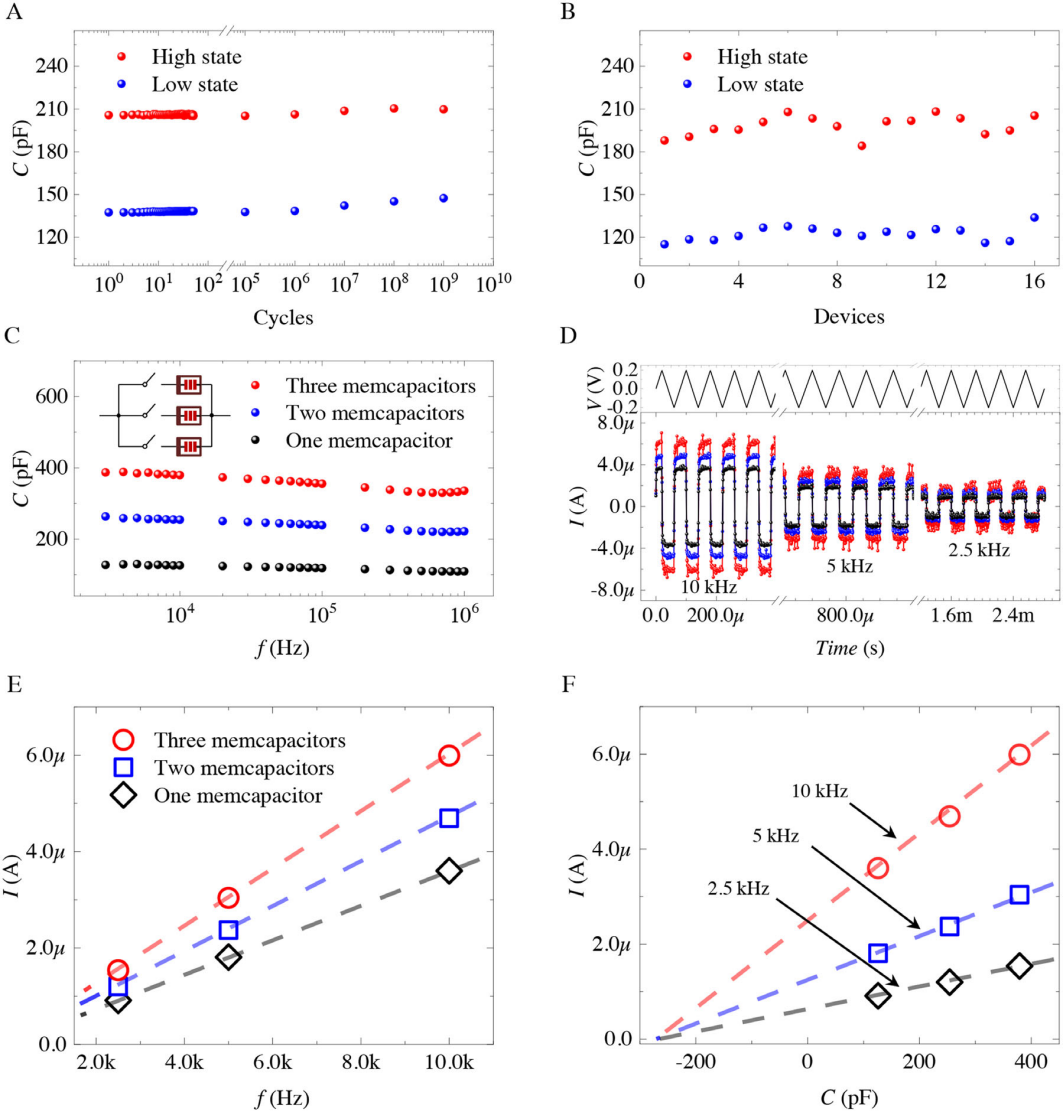
A) The cycle‐to‐cycle and B) device‐to‐device variation of high and low capacitance states for metal/ferroelectric/metal/insulator/semiconductor (MFMIS) memcapacitor with 120 nm P(VDF‐TrFE) and 25 nm SiO_2_. C) The frequency dependence of capacitance value for one, two, and three parallel ferroelectric memcapacitors, respectively. D) Evolution of displacement currents of one, two, and three parallel ferroelectric memcapacitors under triangular voltage signals with a frequency of 10, 5, and 2.5 kHz, respectively. The linear relationship for E) *I* versus d*V*/d*t* (E) and F) *I* versus *C*.

The cycle‐to‐cycle variation and device‐to‐device variation in these devices have been evaluated. Evolution of *C*–*V* hysteresis loops under endurance cycles in an MFMIS memcapacitor with 120 nm‐thick P(VDF‐TrFE) and 25 nm‐thick SiO_2_ is checked (Figure S4). Amazingly, the device shows robust endurance behavior and only suffers a slight variation even after 10^9^ triangular wave cycles (Figure 4A). To evaluate the device‐to‐device variation, the *C*–*V* hysteresis loops for 16 different MFMIS‐based memcapacitors with 120 nm‐thick P(VDF‐TrFE) and 25 nm‐thick SiO_2_ are collected in Figure S5. Figure 4B shows the high capacitance states and low capacitance states in these 16 distinct memcapacitors, showing low device‐to‐device variations that may originate from our rudimentary film deposition technique. Indeed, for example, the film morphologies and domain structures usually have a big influence on the electrical performance.^[^ ^30^ ^]^ The relatively stable capacitance distribution among all these devices can be attributed to the homogeneity of our samples and renders feasible to design memcapacitive crossbar array.

The robust and reconfigurable memcapacitive states guarantee that the memcapacitor would be a promising device for in‐memory computing. The basis for parallel computation in ANNs is the VMM operations. For example, the memcapacitance states can be distinguished by reading the displacement currents (*I* = *C* × d*V*/d*t*) under a designed triangle voltage waveform whose amplitude is much smaller than the coercive voltage of the ferroelectric so that these memcapacitive states remain stable during the reading process. In the ferroelectric memcapacitor crossbar array, to achieve the VMM operation based on the formula of *I* = *C* × d*V*/d*t*, it also requires that the capacitance value is independent on frequency of testing signals (d*V*/d*t*) in a large frequency range. Figure 4C gives the frequency dependence of the capacitance value for one, two, and three parallel ferroelectric memcapacitors, respectively. It shows that the capacitance value slightly depends on frequency of testing signals in a large frequency range from 1 kHz to 1 MHz. The evolution of displacement currents of one, two, and three parallel ferroelectric memcapacitors under triangular voltage signals with a frequency of 10, 5, and 2.5 kHz, respectively, has been further checked as shown in Figure 4D. As expected, the availability of formula of *I* = *C* × d*V*/d*t* is experimentally confirmed by the linear relationship for both *I* versus d*V*/d*t* (Figure 4E) and *I* versus *C* (Figure 4F). The crossover at − 266 pF in Figure 4F implies the existence of series of parasitic capacitance likely originating from the testing circuit, for example between electric connections and between core and shield of the long electric wires. By using the programable frequency‐independent capacitance states as weight, the linear relationship for both *I* versus *C* and *I* versus d*V*/d*t* allows the in‐memory computing application in which VMM operations can be achieved accounting the formula of *I* = *C* × d*V*/d*t*.

**FIGURE 5:**
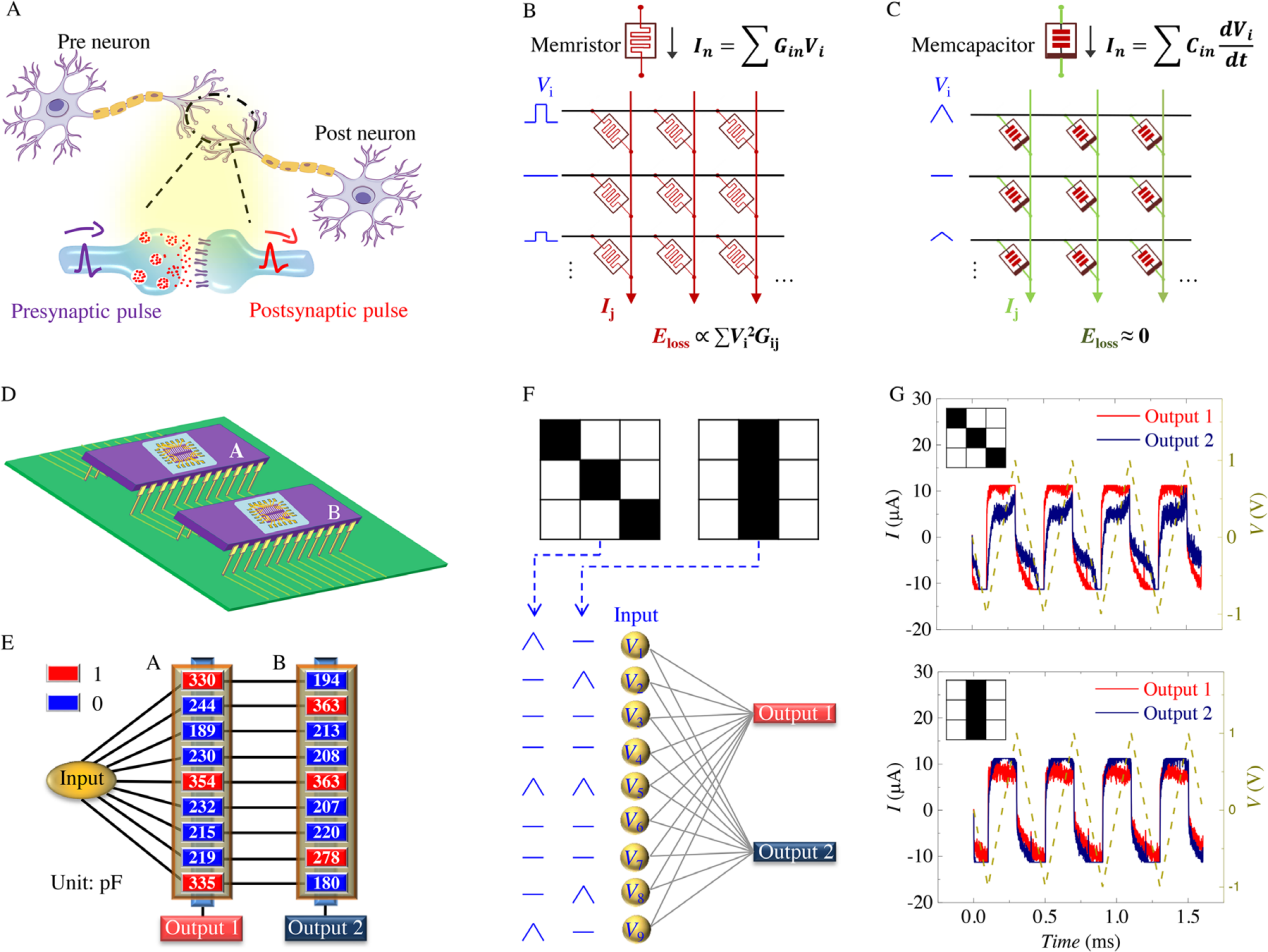
Pattern recognition using a ferroelectric memcapacitor crossbar array. A) Illustration of two neurons connected through a synapse. B) Currents flow in a memristor crossbar array. C) Currents flow in a memcapacitor crossbar array. D) Depiction of our wired 9 × 2 memcapacitor crossbar array. E) Network arrangement of the 9 × 2 memcapacitor crossbar array. F) Flow chart of the inference process to classify the diagonal pixel pattern and vertical pixel pattern. G) The acquisition of displacement currents by inputting the diagonal pixel pattern and vertical pixel pattern to our ferroelectric memcapacitor crossbar array.

Inspired by the massively parallel computing in a biological brain, in which various information is achieved through modulating the synaptic weights between connected neurons (Figure 5A), memristor crossbar arrays, where the memristor plays the role of the artificial synapse, have been proposed to implement energy‐efficient in‐memory computing. In a memristor crossbar array, synaptic weights are achieved by the tunable conductance states. As sketched in Figure 5B, memristor devices are located at each cross point of the crossbar array. Based on the Ohm's and Kirchhoff ’s laws, each column current is a summation of that through all memristors in this column, while the current value through each memristor equals the multiplication of the voltage bias and its conductance.^[^ ^31^, ^32^ ^]^ The column currents obey the formula: $I_{n}=\sum_{i}G_{\mathrm{in}}\;V_{i}$. Through encoding the input vector by different amplitudes of input voltage pulse and the matrix content by the analog memristor conductance, VMM results can be directly read from these output column currents. Despite recent progresses has proven the feasibility of the memristive crossbar arrays,^[^ ^33^ ^]^ analog storage of synaptic weights in a manner of conductance inevitably generates energy dissipation through Joule heating, which also impedes the very‐large‐scale and highly complicated hardware integration as in the human brain.

Alternatively, the ferroelectric‐based memcapacitor can offer a solution to circumvent Joule heating issues. Memcapacitive states can be read out through displacement currents as illustrated in Figure 4D. Likewise, the memcapacitive crossbar array is constituted to achieve the VMM operations using the electrical formula, that is, $I_{n}=\sum_{i}C_{\mathrm{in}}\;\frac{dV_{i}}{dt}$. As schematically shown in Figure 5C, through encoding the input vector by different slopes ($\frac{dV_{i}}{dt}$) of input triangular voltage and the matrix content by the analog memcapacitor capacitance, VMM results can be directly read from collected displacement currents in each column. In principle, during the acquisition of displacement current, the memcapacitor itself does not produce any Joule heating at all!

To confirm the suitability of the proposed memcapacitor crossbar array, two ferroelectric memcapacitor arrays are wired together on a printed circuit board to form a 9 × 2 crossbar network (Figure 5D). Note that the ferroelectric memcapacitor crossbar array can be effectively programmed without suffering from sneak path issue because of the threshold effect of polarization reversal.^[^ ^29^ ^]^ We define that the “0” corresponds to a low capacitance value of around 200 pF and the “1” stands for a high capacitance value of around 300 pF. The capacitance value in the ferroelectric memcapacitor crossbar array is preprogrammed as “100010001” and “010010010” in two columns, respectively (Figure 5E). This ferroelectric memcapacitor crossbar array is used to classify two 9‐pixel patterns, a diagonal one and a vertical one (Figure 5F). The 9 pixels in each pattern are encoded by a triangular voltage waveform as input signals. Black and white pixels represent a constant and null triangular voltage waveform, respectively. Then both the diagonal pattern and vertical pattern are input to the ferroelectric memcapacitor crossbar array and the displacement currents from the two columns are collected. As presented in Figure 5G, when executing the diagonal (vertical) pattern, Output 1 (Output 2) representing the “100010001” (“010010010”) column gives a larger displacement current signal, implying a successful pattern recognition using our memcapacitive VMM operations.

## 3 CONCLUSION

In summary, we demonstrate the memcapacitive behavior in ferroelectric‐based both MFIS and MFMIS structures. More than 3‐bit reconfigurable capacitance states are obtained in an MFMIS memcapacitor. These robust memcapacitive states demonstrate a long retention time of more than 10^4^ s and a good endurance of 10^9^ cycles. The memcapacitive VMM operations are realized using the electrical formula *I* = *C* × d*V*/d*t* and Kirchhoff's current law. Through encoding the input vector by different slopes ($\frac{dV_{i}}{dt}$) of input triangular voltage and the matrix content by the analog memcapacitor capacitance, VMM results can be directly read from collected displacement currents in each column of the memcapacitor crossbar array. Based on the proposed memcapacitive VMM operations, two 9‐pixel patterns are successfully recognized in our wired 9 × 2 MFMIS‐structure ferroelectric memcapacitor crossbar network hardware. In principle, the memcapacitor itself does not generate any Joule heating during the displacement currents‐involved inference process. This study unveils ferroelectric memcapacitors with an in‐memory computing paradigm, opening up a new route for the development of reliable and ultralow‐power neural hardware.

## 4 EXPERIMENTAL SECTION

### 4.1 Preparation of ferroelectric memcapacitors

First, the p‐type silicon wafer (*ρ* = 1 ∼ 10 Ω•cm) with 25 nm SiO_2_ was cleaned using acetone, ethanol, and deionized water for 10 min, respectively. 100 nm‐thick ferroelectric P(VDF‐TrFE) films were then spin‐coated (2500 rpm for 30 s) on the substrate and annealed at 135°C for 4 h. Finally, 50‐nm top Al electrodes were deposited through vacuum thermal evaporation assisted by a metal mask to form the MFIS memcapacitor. The inserted Au metal layer (50 nm) in the MFMIS memcapacitor is also deposited through vacuum thermal evaporation assisted by a metal mask.

### 4.2 Electrical measurement

Before electrical tests of memcapacitors, the silicon terminal is grounded. *C*–*f*, *C*–*V*, *C*–*t*, and pattern recognition data were measured and collected using a semiconductor parameter analyzer (Keithly S4200A). LTP and LTD tests were carried out with an Agilent E4980A LCR meter. All capacitance value is measured under the ac voltage signal with an amplitude of 0.2 V and a frequency of 1 kHz after removing the biased voltage pulses.

## CONFLICT OF INTEREST STATEMENT

The authors declare no conflicts of interest.

## Supporting information

Supporting Information

## Data Availability

The data that support the findings of this study are available from the corresponding author upon reasonable request.
