# Supplementary material for: Ultralow‐power in‐memory computing based on ferroelectric memcapacitor network
Source: Exploration (Beijing). 2023 May 11;3(3):20220126. doi: 10.1002/EXP.20220126 (PMC10624373; doi:10.1002/EXP.20220126)
Supplement: Supplementary file 1 — Supporting Information [file EXP2-3-20220126-s001.pdf]

Supporting information

Ultralow-power in-memory computing based on ferroelectric memcapacitor network

Bobo Tian[1,2], Zhuozhuang Xie[1,3], Luqiu Chen[1], Shenglan Hao[1,4], Yifei Liu[1], Guangdi Feng[1], Xuefeng Liu[1], Hongbo Liu[3], Jing Yang[1], Yuanyuan Zhang[1], Wei Bai[1], Tie Lin[5], Hong Shen[5], Xiangjian Meng[5], Ni Zhong[1], Hui Peng[1], Fangyu Yue[1], Xiaodong Tang[1], Jianlu Wang[6], Qiuxiang Zhu[1,2,8], Yachin Ivry[9], Brahim Dkhil[4], Junhao Chu[1,5,7] and Chungang Duan[1,10]

1 Key Laboratory of Polar Materials and Devices, Ministry of Education, Shanghai Center of Brain-inspired Intelligent Materials and Devices, East China Normal University, Shanghai 200241, China

2 Zhejiang Lab, Hangzhou 310000, China.

3 School of Materials Engineering, Shanghai University of Engineering Science, Shanghai, 201620, China

4 Université Paris-Saclay, CentraleSupélec, CNRS-UMR8580, Laboratoire SPMS, 91190 Gif-sur-Yvette, France.

5 State Key Laboratory of Infrared Physics, Shanghai Institute of Technical Physics, Chinese Academy of Sciences, 500 Yu Tian Road, Shanghai 200083, China.

6 Frontier Institute of Chip and System, Fudan University, 220 Handan Road, Shanghai, 200433, China.

7 Institute of Optoelectronics, Fudan University, 220 Handan Road, Shanghai, 200433, China.

8 Guangdong Provisional Key Laboratory of Functional Oxide Materials and Devices, Southern University of Science and Technology, Shenzhen 518055, China.

9 Department of Materials Science and Engineering, Solid-State Institute, Technion-Israel Institute of Technology, Haifa, 3200003 Israel.

10 Collaborative Innovation Center of Extreme Optics, Shanxi University, Shanxi 030006, China.

Correspondence

Bobo tian and Qiuxiang Zhu, Key Laboratory of Polar Materials and Devices, Ministry of Education, Shanghai Center of Brain-inspired Intelligent Materials and Devices, East China Normal University, Shanghai 200241, China.

Email: bbtian@ee.ecnu.edu.cn and qxzhu@clpm.ecnu.edu.cn

Hongbo Liu, School of Materials Engineering, Shanghai University of Engineering Science, Shanghai, 201620, China.

Email: liuhongbo@sues.edu.cn

Bobo Tian, Zhuozhuang Xie, and Luqiu Chen contributed equally to this work.

FIGURES

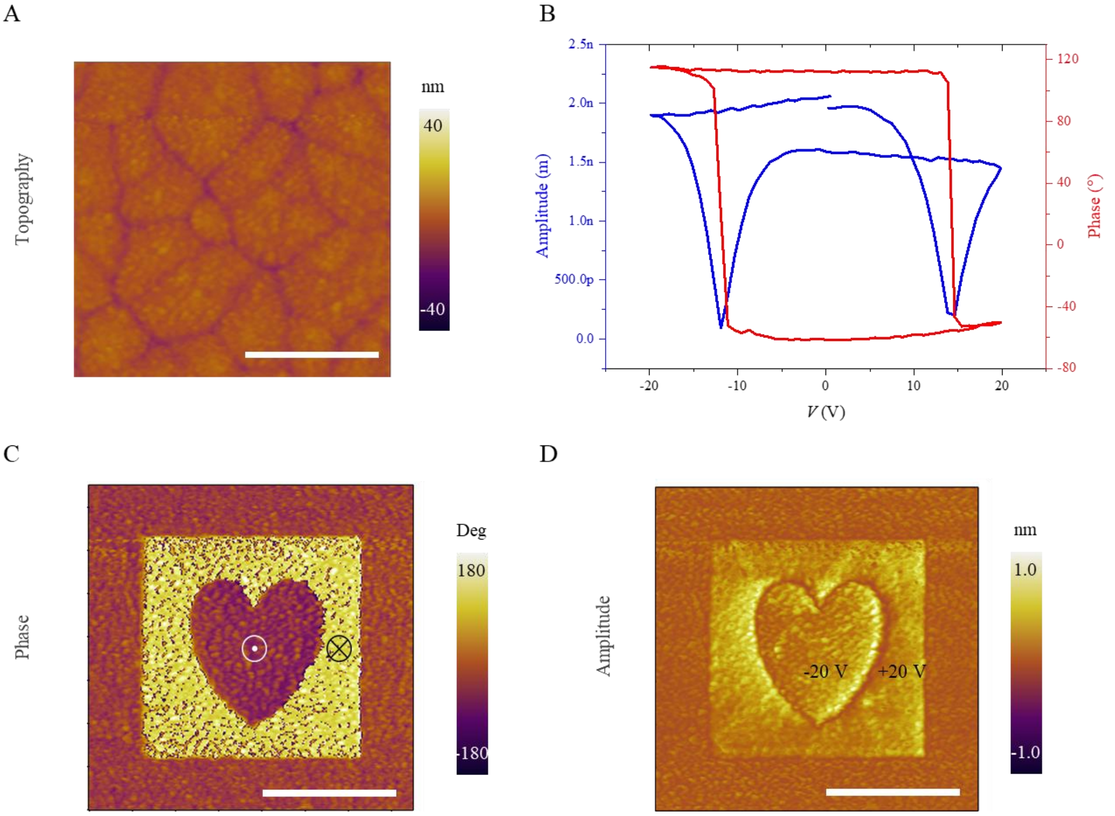

FIGURE S1 A) AFM topography image of P(VDF-TrFE) thin films. B) PFM Piezoelectric hysteresis loops of P(VDF-TrFE) thin films: phase (red) and amplitude (lue). C) PFM phase and D) amplitude image of P(VDF-TrFE) films after writing a square area on film surface with a positive voltage (+20 V) followed by a smaller "heart" with a negative voltage (-20 V) by a biased conductive tip. The scale bar is 6 µm.

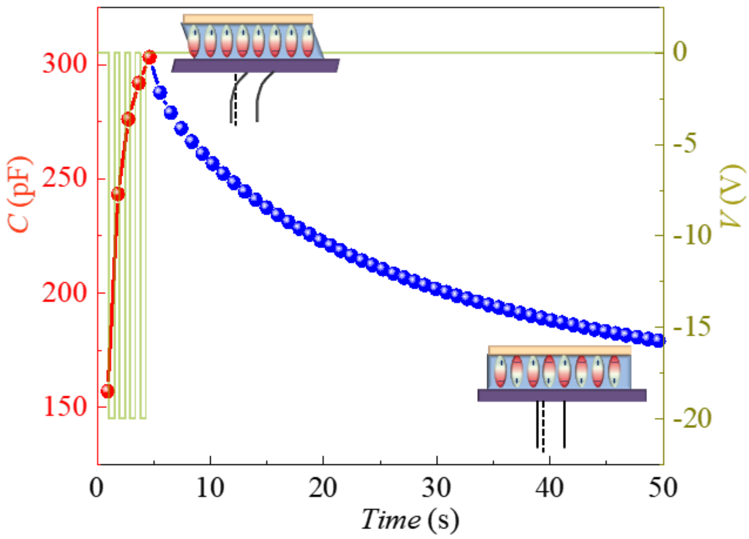

FIGURE S2 Retention of the programmed high capacitance state in the MFIS memcapacitor with 60 nm-thick P(VDF-TrFE) and 25 nm-thick SiO_2.

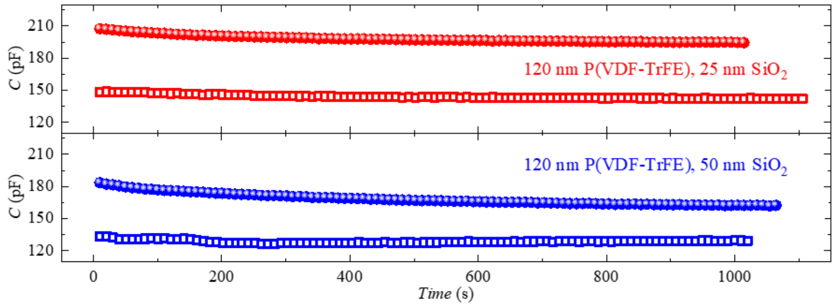

FIGURE S3 Retention of the highest capacitance state and the lowest capacitance state in a MFMIS memcapacitor with 120 nm-thick P(VDF-TrFE) and 25 nm-thick (red) or 50 nm-thick (blue) SiO_2.

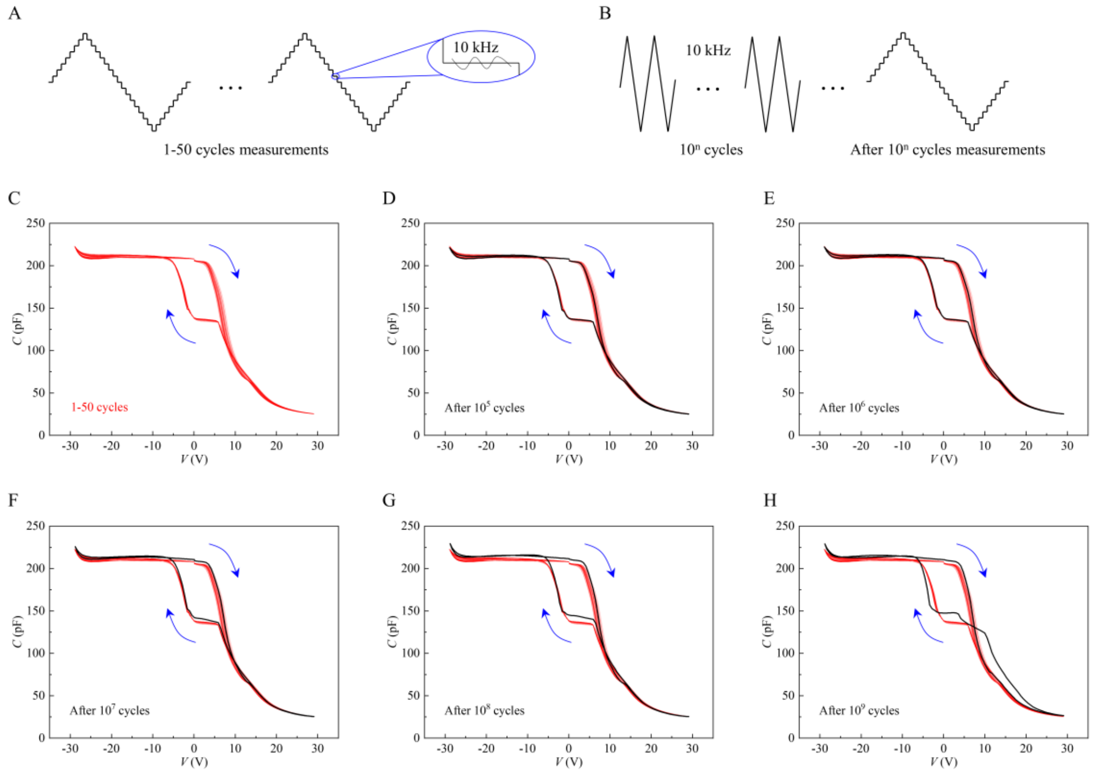

FIGURE S4 Evolution of *C*-*V* hysteresis loops under endurance cycles in a MFMIS memcapacitor with 120 nm-thick P(VDF-TrFE) and 25 nm-thick SiO_2. The amplitude of triangular wave cycles in (D-H) is 30 V, which is larger than the coercive voltage in the MFMIS memcapacitor.

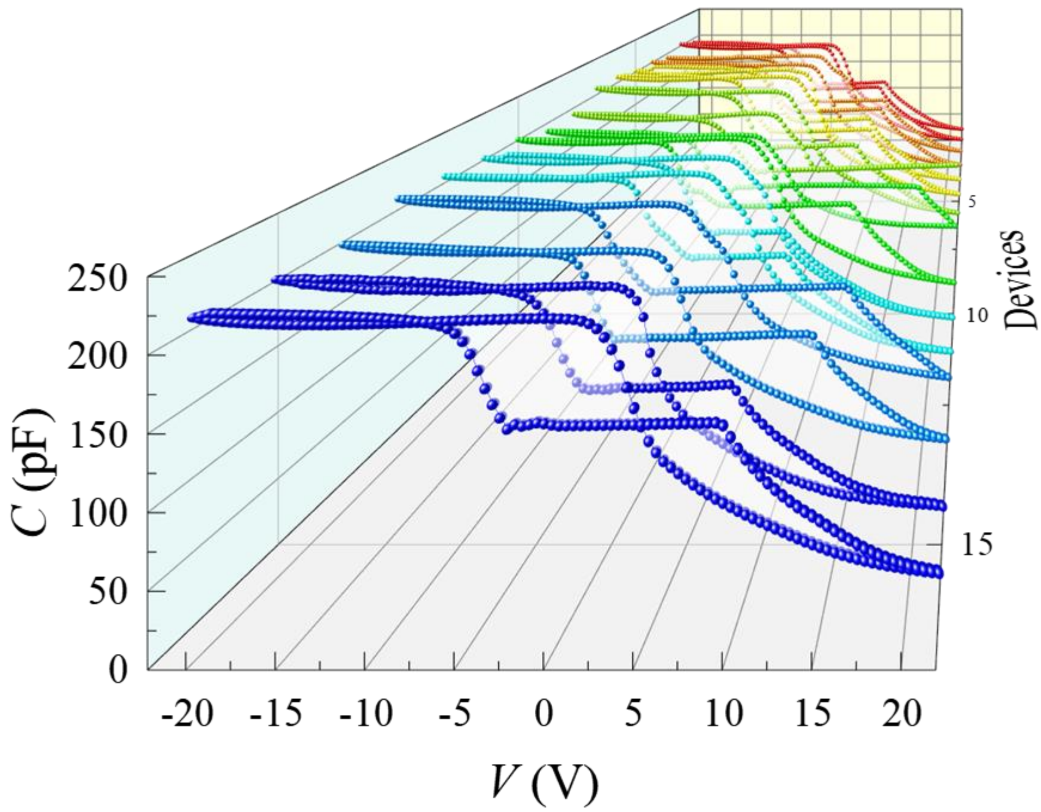

FIGURE S5 *C*-*V* hysteresis loops for 16 different MFMIS memcapacitors with 120 nm-thick P(VDF-TrFE) and 25 nm-thick SiO_2.
